# Colossal emergent inductance in a molecular memristor

**DOI:** 10.1038/s41598-026-48808-5

**Published:** 2026-05-08

**Authors:** Yugo Oshima, Rei Usami, Tetsuro Moriya, Taishi Takenobu, Shinya Takaishi

**Affiliations:** 1 RIKEN, Pioneering Research Institute (RIKEN-PRI), Hirosawa 2-1, Wako-shi, Saitama 351-0198 Japan; 2 Department of Applied Physics, Nagoya University, Furo-cho, Chikusa-ku, Nagoya, 464-8603 Japan; 3 Department of Chemistry, Graduate School of Science, Tohoku University, Aza-aoba, Aramaki, Sendai, 980-8578 Japan

**Keywords:** Memristor, Mott insulator, Emergent inductance, Correlated electron materials, Nonlinear electronic response, Functional electronic materials, Engineering, Materials science, Nanoscience and technology, Physics

## Abstract

Memristors exhibit history-dependent transport and are widely studied for their hysteretic current–voltage characteristics, yet their dynamical electrodynamic roles remain largely unexplored. Here, we investigate the quasi-one-dimensional halogen-bridged metal complex [Ni(chxn)$_2$Br]Br$_2$ using transport measurements, impedance spectroscopy, and oscillation analysis. We show that this material functions as a memristor exhibiting a clear pinched hysteresis loop (PHL) under *ac* bias. Remarkably, this hysteresis gives rise to a colossal emergent inductance of $10^4$–$10^5$ H, far exceeding that of conventional coil-based inductors. The inductive response appears only under finite bias, ruling out parasitic origins, and is independently confirmed by impedance spectroscopy and oscillation-frequency analysis. When combined with a simple capacitor, this intrinsic inductance together with negative differential resistance drives self-sustained oscillations without any external inductor, redefining the origin of oscillatory behavior in this system. These results establish emergent inductance as a fundamental memristive property, reveal a new electrodynamic functionality in correlated molecular materials, and suggest potential routes toward coil-free low-frequency functionalities in electronic and neuromorphic systems.

## Introduction

Memristors are nonlinear passive electronic components whose resistance depends on the history of charge flow^1,2^. This property gives rise to a characteristic pinched hysteresis loop (PHL) in the current-voltage (*I*-*V*) response under periodic driving^2^. Such hysteretic transport has attracted broad interest in the contexts of non-volatile memory, neuromorphic computing, and nonequilibrium dynamics^3–5^.

Recently, we reported that the bilayer-type nickel-dithiolene complex (Et-4BrT)[Ni(dmit)$_2$]$_2$ exhibits nonlinear transport with negative differential resistance (NDR) and a clear PHL under an applied *ac* bias, thereby establishing it as a memristor^6^. Here, Et-4BrT denotes ethyl-4-bromothiazolium and dmit stands for 1,3-dithiole-2-thione-4,5-dithiolate. Importantly, we demonstrated that a large inductive response can dynamically emerge from memristive hysteresis under bias, reaching values of order $10^2$ H. When coupled to a simple external capacitor, this emergent inductance drives self-sustained oscillations without any discrete inductor—a phenomenon we termed memristive oscillation. These results revealed that memristors can function not only as oscillators but also as intrinsic sources of inductance.

Motivated by this finding, we turned to the quasi-one-dimensional (q1D) halogen-bridged metal complex [Ni(chxn)$_2$Br]Br$_2$(chxn = cyclohexanediamine), a molecular Mott insulator long known to exhibit NDR and relaxation-like self-oscillation when shunted by a capacitor^7–12^. Despite extensive study, its oscillatory behavior has conventionally been interpreted within a relaxation-oscillator framework that does not invoke any inductive element^8^. We targeted [Ni(chxn)$_2$Br]Br$_2$ because its large insulating resistance is expected to strongly enhance any emergent inductance, and because the unusually low frequency of its self-oscillation already hints at a colossal inductive response. If the link between PHL and inductance identified in (Et-4BrT)[Ni(dmit)$_2$]$_2$ is universal, then capacitor-driven oscillation in [Ni(chxn)$_2$Br]Br$_2$ without an explicit inductor strongly suggests a memristive origin.

Here, we show that [Ni(chxn)$_2$Br]Br$_2$ is in fact a molecular Mott memristor exhibiting a pronounced PHL and a colossal emergent inductance exceeding 100 kH—many orders of magnitude larger than conventional coil-based inductors and far greater than that observed in (Et-4BrT)[Ni(dmit)$_2$]$_2$. This colossal inductance not only redefines the origin of oscillation in this prototypical q1D system but also opens perspectives for low-frequency filtering, timing, and neuromorphic device applications. By combining impedance spectroscopy and oscillation-frequency analysis, we quantitatively confirm the intrinsic nature and magnitude of this inductive response, excluding extrinsic circuit artifacts.

## Results

### Pinched hysteresis loop as a signature of memristive dynamics

**Fig. 1:**
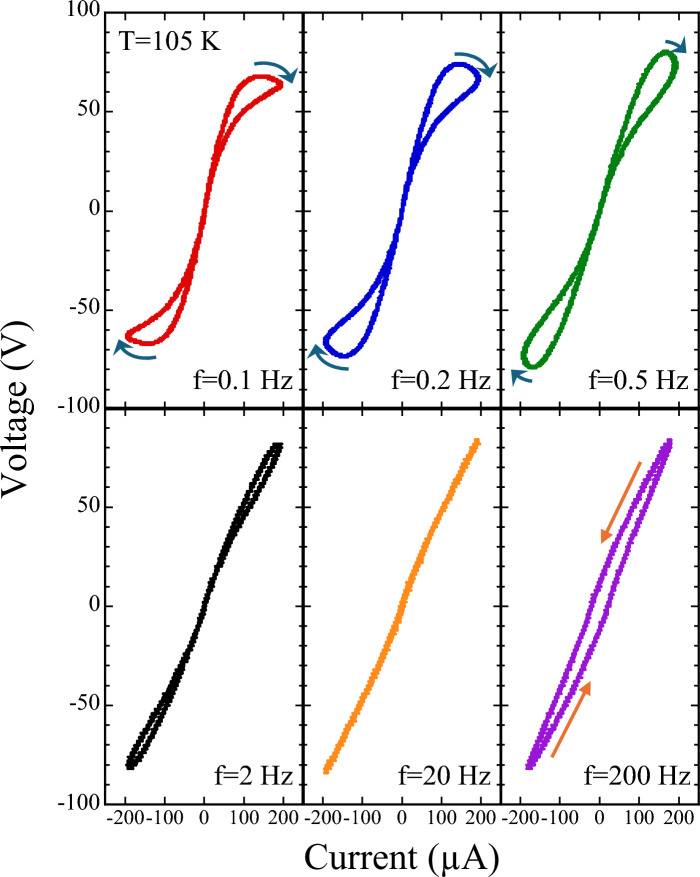
Frequency dependence of Lissajous curves for [Ni(chxn)$_2$Br]Br$_2$ at 105 K. At low frequencies (0.1–0.5 Hz), clear pinched hysteresis loops (PHLs) with negative differential resistance (NDR) are observed. As the frequency increases, the NDR feature disappears and the loops gradually collapse into linear traces (0.5–20 Hz). At high frequencies (200 Hz), the curves evolve into ellipses with a phase shift, reflecting capacitive reactance behavior.

To verify whether [Ni(chxn)$_2$Br]Br$_2$ is indeed a memristor, we first examined its *ac* *I*-*V* response in the form of Lissajous curves. A sinusoidal *ac* current with a peak amplitude of 200 $\mu$A was applied along the chain axis, and the resulting *ac* voltage across the sample was recorded. Figure 1 shows the frequency dependence of the Lissajous curves for [Ni(chxn)$_2$Br]Br$_2$ at 105 K. At low frequencies (0.1–0.5 Hz), clear PHLs accompanied by NDR are observed. The NDR region is indicated by the blue curved arrow in the figure. As the frequency increases, the NDR feature disappears and the loops gradually collapse into nearly linear traces (0.5–20 Hz). At higher frequencies (200 Hz), the curves evolve into counterclockwise ellipses, indicating a phase shift between current and voltage that reflects capacitive reactance behavior. This systematic evolution—from low-frequency PHLs with NDR to nearly linear *I*-*V* traces at intermediate frequencies—is a hallmark of memristive dynamics, while the counterclockwise capacitive loops observed at very high frequencies mainly reflect the intrinsic capacitance of the high-resistance sample^2^.

**Fig. 2:**
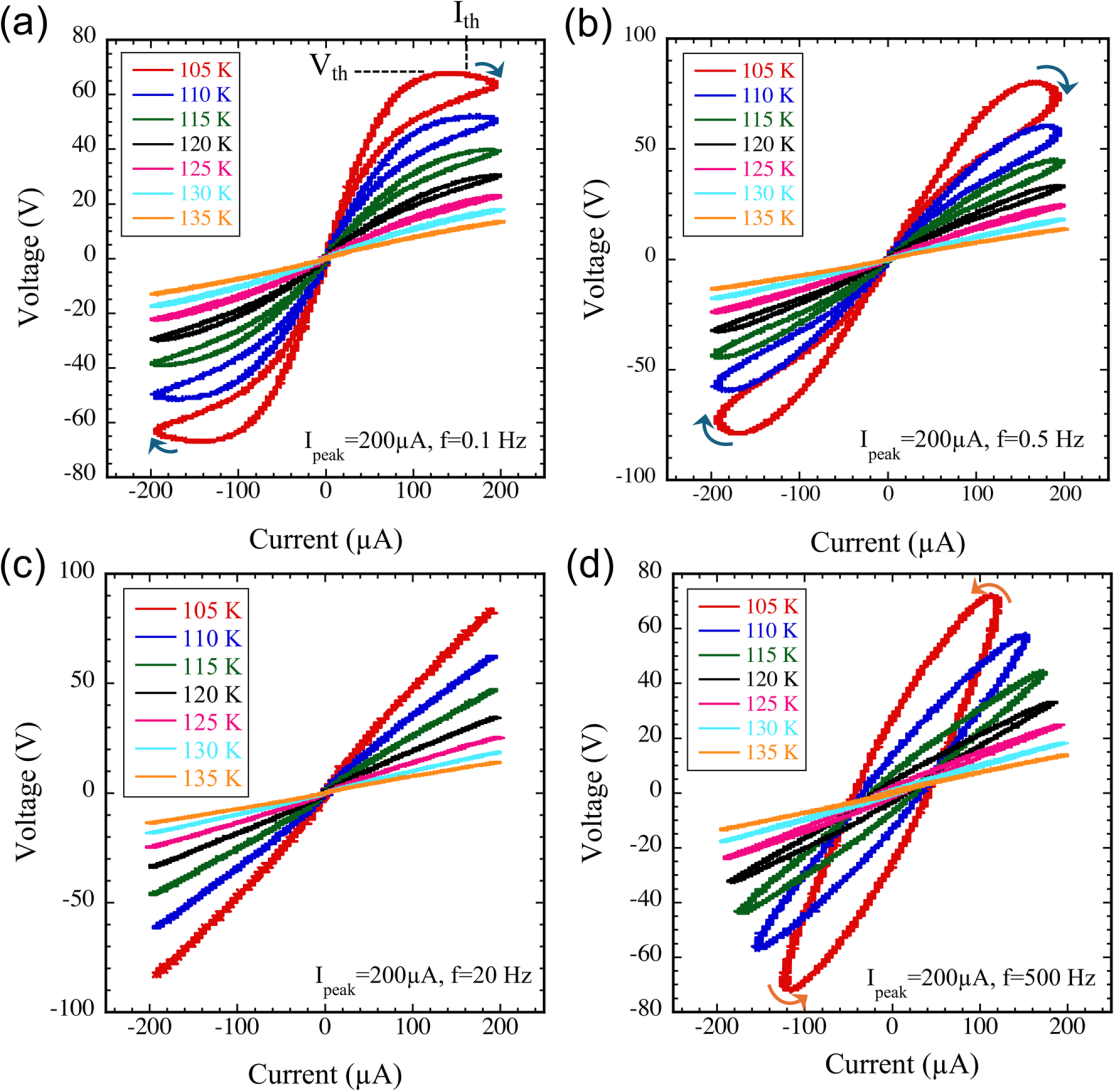
Lissajous curves of [Ni(chxn)$_2$Br]Br$_2$ measured between 105 and 135 K at selected frequencies: (**a**) 0.1 Hz, (**b**) 0.5 Hz, (**c**) 20 Hz, and (**d**) 500 Hz. A sinusoidal *ac* current with a peak amplitude of 200 $\mu$A was applied along the chain axis. The blue curved arrow serves as a visual guide indicating the NDR region. $V_{th}$ and $I_{th}$ denote the threshold voltage and current for entering into the NDR regime, respectively.

To further disentangle the temperature and frequency effects, we next examined the temperature dependence of the Lissajous curves at selected frequencies. The Lissajous curves of [Ni(chxn)$_2$Br]Br$_2$ measured between 105 and 135 K at 0.1, 0.5, 20, and 500 Hz are shown in Fig. 2(a), (b), (c), and (d), respectively. Due to instrumental limitations of the current source, measurements below 105 K were not possible.

Figure 2(a) shows the representative case at 0.1 Hz, where at the lowest temperature (105 K) a clear PHL accompanied by NDR is observed. As the temperature increases, the NDR behavior gradually disappears, and the loop shrinks, ultimately collapsing into a linear *I*-*V* trace. This transition is accompanied by a reduction in the slope of the *I*-*V* curve, indicating a decrease in resistance, consistent with the known negative temperature coefficient of resistance in [Ni(chxn)$_2$Br]Br$_2$ (see also Fig. S1(c))^12^.

At 0.5 Hz (Fig. 2(b)), a similar PHL is observed at low temperature but with a less pronounced NDR segment; again, the loop progressively closes and becomes linear with increasing temperature. At 20 Hz (Fig. 2(c)), the *ac* *I*-*V* response is nearly linear already at 105 K, and the slope simply decreases with temperature. At 500 Hz (Fig. 2(d)), the curves form counterclockwise ellipses that also shrinks as the temperature rises.

A closed loop in the Lissajous curve reflects a phase shift between the *ac* current and voltage, indicative of reactive components in the system. Notably, the PHL observed at low frequencies rotates clockwise (Figs. 2(a) and (b)), whereas the high-frequency elliptical loops rotate counterclockwise (Fig. 2(d)), suggesting the presence of both inductive and capacitive reactance. Above 135 K, no PHL is observed at any frequency, indicating that the memristive behavior is lost at higher temperatures.

### Colossal emergent inductance revealed by impedance spectroscopy

The clockwise and counterclockwise closed loops observed in the Lissajous curves suggest the coexistence of inductive and capacitive reactance in the system. To quantitatively evaluate these reactive components—particularly the emergent inductance—we next performed impedance spectroscopy. This technique provides access to the complex frequency-dependent response, allowing us to extract effective circuit parameters including resistance, capacitance, and inductance^13^.

**Fig. 3:**
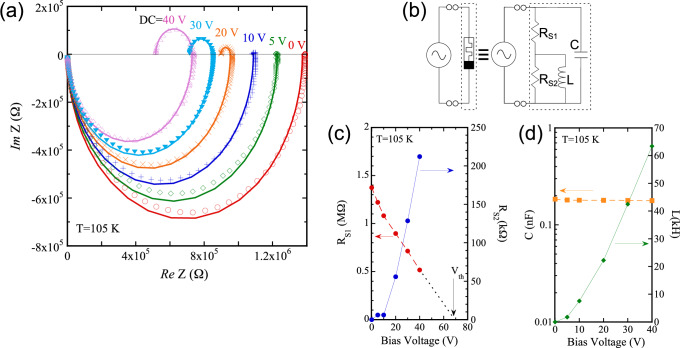
(**a**) Cole–Cole plots of the impedance spectra at 105 K for selected *dc* bias voltages (colored dots) and corresponding fitted curves (solid lines), using the equivalent circuit shown in (**b**). The applied *ac* voltage was 3 V, and the frequency range was from 10 mHz to 67.2 kHz. (**c**) $R_{S1}$ and $R_{S2}$, and (**d**) *C* and *L* as functions of $V_{bias}$, extracted from the fitting in (**a**).

Figure 3(a) shows the Cole-Cole plots of [Ni(chxn)$_2$Br]Br$_2$ at 105 K. A sinusoidal *ac* voltage of 3 V was applied, and *dc* bias voltage ($V_{bias}$) was varied from 0 to 40 V during the measurement. The *ac* voltage amplitude was chosen to ensure sufficient signal-to-noise ratio in the high-impedance regime, where smaller excitation signals do not yield reliable impedance spectra or well-defined semicircular features in the Cole–Cole plots. At $V_{bias}$ = 0 V, the Cole-Cole plot exhibits only a negative semicircle in the imaginary part of impedance, corresponding to capacitive reactance. Upon applying a finite $V_{bias}$, a positive semicircle emerges in the upper half-plane, indicating inductive reactance. Notably, the inductive semicircle emerges only under finite bias and is absent at $V_{bias}$ = 0, which excludes trivial contributions from parasitic inductance of leads or external components. This behavior closely resembles that observed in our previous study on the molecular memristor (Et-4BrT)[Ni(dmit)$_2$]$_2$^6^. In both cases, the inductive semicircle shifts toward lower resistance with increasing $V_{bias}$.

To capture the emergent inductive response, we analyzed the Cole–Cole plots using an equivalent circuit model consisting of two resistive channels ($R_{S1}$+$R_{S2}$), a capacitance (*C*), and an inductance (*L*) connected in series and parallel as shown in (b). This model was previously validated for the molecular memristor (Et-4BrT)[Ni(dmit)$_2$]$_2$, and is sufficient to reproduce both the capacitive and inductive arcs observed in the spectra^6^. The fitted curves are overlaid in Fig. 3(a), and the extracted parameters ($R_{S1}$, $R_{S2}$, *C* and *L*) are summarized in Fig. 3(c) and (d). A detailed description of the Cole-Cole characteristics of various equivalent circuits is provided in the Supporting Information (SI).

As explained in the SI, the left intercept of the inductive semicircle corresponds to the low-frequency limit of the real impedance, representing the slope resistance $R_{S1}$. The right intercept indicates the high-frequency limit, corresponding to the static resistance ($R_{S1}$+$R_{S2}$) (see also Fig. S2(b)). As $V_{bias}$ increases, the slope resistance, $R_{S1}$, decreases monotonically (Fig. 3(c)), consistent with the progressive flattening of the gradient in the Lissajous curve. Although the measurement was limited to $V_{bias}$ = 40 V, extrapolation suggests that $R_{S1}$ approaches zero near $V_{bias}$ $\approx$ 70 V. This value agrees with the threshold voltage $V_{th} \approx$ 70 V observed in the Lissajous curve (Fig. 2(a)), where the slope of the PHL flattens to zero (i.e. zero slope resistance) and the system enters the NDR regime.

Even below this threshold $V_{th}$, however, the Cole–Cole plots (Fig. 3(a)) already exhibit a positive semicircle in the upper half-plane, indicating the emergence of inductive reactance. This implies that the inductive response does not simply originate from static NDR, but rather from the dynamic hysteresis represented by the PHL. In other words, once the system is driven into the closed-loop regime of the PHL under finite bias, the *ac* response acquires an effective inductance.

The capacitance *C* remains nearly constant at $\sim$ 0.1 nF across all bias voltages. In contrast, the inductance *L* increases significantly with $V_{bias}$, reaching a colossal value of $\sim$63 kH at $V_{bias}$ = 40 V (Fig. 3(d)). A similar bias-induced inductance was observed in (Et-4BrT)[Ni(dmit)$_2$]$_2$, but the magnitude of *L* in the present compound is two orders of magnitude larger^6^.

**Fig. 4:**
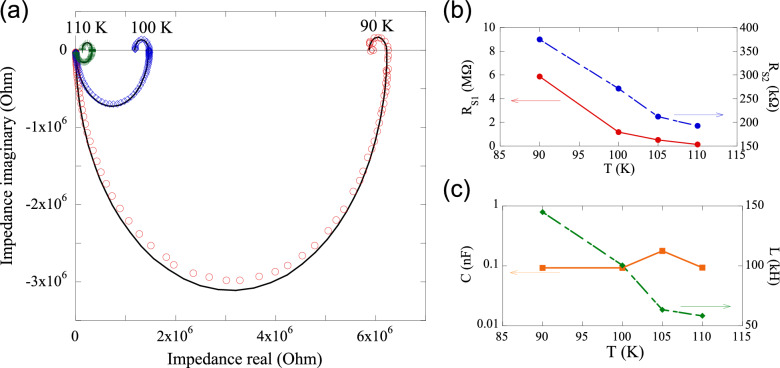
(**a**) Temperature dependence of the Cole–Cole plots at $V_{bias}$ = 40 V, with corresponding fitted curves using the same equivalent circuit as in Fig. 3(**b**) The applied *ac* voltage was 3 V, and the frequency range was from 10 mHz to 67.2 kHz. (**b**) $R_{S1}$ and $R_{S2}$, and (**c**) *C* and *L* as functions of temperature, extracted from the fits in (**a**).

The temperature dependence of the impedance spectra under $V_{bias}$ = 40 V is shown in Fig. 4(a), and the extracted circuit parameters are plotted in Fig. 4(b) and (c). As expected from transport measurements, $R_{S1}$ increases steeply with decreasing temperature (Fig. 4(b)), making it progressively harder to reach the NDR regime. While *C* remains nearly unchanged, *L* increases with decreasing temperature, reaching a maximum inductance of $\sim$145 kH at 90 K (Fig. 4(c)).

### Self-sustained oscillation driven by memristive inductance

To independently validate the colossal inductance extracted from impedance spectroscopy, we next investigated whether it could also be quantified from self-sustained oscillations. In this approach, the oscillation frequency directly reflects the effective inductance when the device is connected in parallel with an external capacitor.

**Fig. 5:**
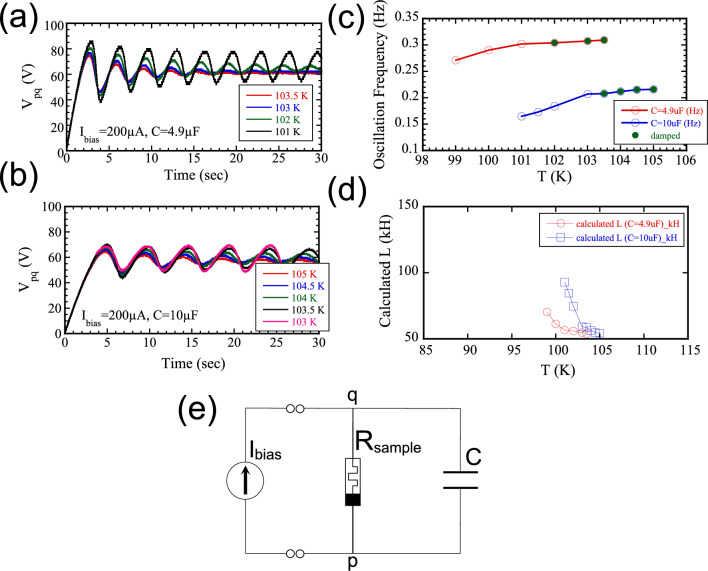
Temperature dependence of memristive oscillation for $I_{bias}$= 200 $\mu$A, with external capacitance of (**a**) 4.9 $\mu$F and (**b**) 10 $\mu$F. Low-temperature data are omitted in both panels for clarity. (**c**) Oscillation frequency as a function of temperature for both capacitance values. (**d**) Inductance *L* estimated from the oscillation frequencies in (**c**) using the *LC* resonance relation. (**e**) Circuit diagram for the memristive oscillation.

Since [Ni(chxn)$_2$Br]Br$_2$ exhibits a PHL with NDR at low frequencies and a colossal inductance under finite $V_{bias}$, it is expected to support memristive oscillation in such a configuration^6^. The circuit diagram for this setup is shown in Fig. 5(e), where a constant *dc* bias current ($I_{bias}$) is applied, and the voltage across the memristor ($V_{pq}$) is monitored.

As shown in Fig. S4(a), self-sustained voltage oscillations appear when $I_{bias}$ exceeds $\sim$160 $\mu$A. This threshold current agrees well with the onset of NDR ($I_{th}$) observed in the Lissajous curves (see Fig. 2(a)), confirming that the system must operate within the NDR regime to sustain oscillation.

Figure 5(a) and (b) show the temperature dependence of memristive oscillations at $I_{bias}$ =200 $\mu$A with external capacitances of *C*=4.9 $\mu$F and 10 $\mu$F, respectively. In both cases, the oscillation frequency slightly increases with temperature, while the oscillation amplitude gradually decreases and becomes damped at higher temperatures. This suppression is consistent with the PHL behavior in Fig. 2, where increasing temperature and frequency lead to the disappearance of NDR, which is essential for sustaining the oscillation.

The temperature dependence of the oscillation frequency for both capacitance values is summarized in Fig. 5(c). A slight deviation is observed between the two data sets, reflecting the *C*-dependence expected from the resonance condition of an *R*–*L*–*C* circuit.

The oscillation frequency *f* can be modeled by the standard *LC* resonance relation:

1 $$\begin{aligned} f=\frac{1}{2\pi }\sqrt{\frac{1}{LC}} \end{aligned}$$

Using this expression, the temperature dependence of the inductance *L* was estimated from the measured frequencies in Fig. 5(c), and the results are plotted in Fig. 5(d). The estimated inductance remains in the range of tens to over a hundred kilohenries, and increases monotonically with decreasing temperature, in good agreement with the impedance spectroscopy results in Fig. 4(c).

The close agreement between the inductance values obtained via two independent methods—impedance spectroscopy and oscillation frequency analysis—strongly supports the conclusion that the observed colossal inductance is not a circuit artifact, but a genuine and essential manifestation of the memristive dynamics. This suggests that such emergent inductance may be a general feature of memristors, inherently linked to their pinched hysteresis behavior, although it has rarely been experimentally quantified.

## Discussion

The present results reveal that the q1D Mott insulator [Ni(chxn)$_2$Br]Br$_2$ exhibits the defining memristive signature—a PHL—together with NDR and a colossal inductive response emerging under finite bias. The PHL provides direct evidence that the resistance depends on the internal state and charge history, revealing intrinsic memristive dynamics. These dynamics enable self-sustained oscillation without any discrete inductor: the device itself supplies a bias-induced effective inductance, and when shunted by a small external capacitor, it oscillates spontaneously. While the operating voltage in the present system is relatively high and the characteristic frequency is relatively low compared to conventional molecular memory devices and electronic components, these features originate from the intrinsic properties of the q1D Mott insulating system. A sufficiently large electric field is required to access the nonlinear transport regime associated with memristive hysteresis, while the slow internal dynamics of the correlated electronic state give rise to the low-frequency response. These intrinsic properties are essential for the emergence of the observed inductive behavior, rather than limitations of the system.

The inductive response appears only under finite bias and is absent at zero bias, immediately ruling out trivial contributions from parasitic inductance of leads or external components, which are typically in the nH–$\mu$H range—orders of magnitude smaller than the observed values. The inductance originates intrinsically from the hysteretic current-voltage response (PHL), and its large magnitude can be qualitatively understood as a result of long relaxation times inherent to this hysteresis, further enhanced by the large resistance in the insulating state and by in-gap states that slow carrier dynamics (as supported by the Arrhenius plot in the SI)^12^. Two independent approaches—impedance spectroscopy and oscillation-frequency analysis—consistently confirm the intrinsic nature and magnitude of the inductance, which increases with bias and decreases with temperature, reaching a colossal 145 kH at 90 K. Such magnitudes are extraordinarily large compared with conventional inductors (typically $\mu$H–mH) and far beyond what any coil design could achieve within a millimeter-sized crystal, underscoring the unique origin of this emergent inductance. Because this inductance is an emergent, bias-dependent quantity arising from nonlinear memristive dynamics, it cannot be directly accessed using conventional small-signal techniques such as LCR meters. In such measurements, inductance is obtained by converting the impedance into a predefined series or parallel equivalent circuit, which is not generally appropriate for a strongly nonlinear system exhibiting NDR, and may lead to unreliable or nonphysical values.

Earlier studies interpreted oscillations in [Ni(chxn)$_2$Br]Br$_2$ using a relaxation oscillator framework, treating the material as a resistance-switching element in parallel with a capacitor^8^. Although the waveform resembles that of a relaxation oscillator, the emergent inductance associated with the memristive PHL plays a crucial role in stabilizing the dynamics and establishing a robust limit cycle. This inductance provides an effective inertia for the current, preventing runaway behavior in the NDR regime and enabling reproducible self-sustained oscillations. We emphasize that while NDR alone can lead to self-sustained oscillations in conventional relaxation oscillators, the present system exhibits clear signatures of inductive dynamics. In particular, impedance spectroscopy reveals a finite inductive component, and the oscillation frequency is quantitatively consistent with the independently extracted inductance. The agreement between these independent approaches cannot be explained within a purely *RC*-based framework and thus provides strong evidence for an intrinsic inductive contribution arising from memristive dynamics.

Although this emergent inductance can support oscillatory dynamics, the intrinsic capacitance of [Ni(chxn)$_2$Br]Br$_2$ is small ($\sim$0.1 nF) as shown in Figs. 3(d) and 4(c). For an inductance of 100 kH, the corresponding characteristic frequency scale ($\sim$50 Hz), as estimated from Eq. (1), lies outside the regime where NDR and a pronounced PHL are observed (see Fig. 2(c)), leading to damped oscillations. Introducing a sufficiently large external capacitor shifts the oscillation frequency into the NDR-sustaining regime, thereby stabilizing self-oscillation. Thus, memristive oscillation arises from the cooperative interplay of emergent inductance and NDR, while the capacitance primarily determines the oscillation timescale.

The linkage between PHL and inductive behavior suggests that such emergent inductance is not unique to [Ni(chxn)$_2$Br]Br$_2$. In our previous work on the bilayer conductor (Et-4BrT)[Ni(dmit)$_2$]$_2$, a similar but smaller inductive response was observed^6^. More broadly, emergent inductance has been reported in correlated and magnetic systems such as Ca$_2$RuO$_4$, Gd$_3$Ru$_4$Al$_{12}$, and YMn$_6$Sn$_6$^14–16^. Although those studies did not explicitly invoke the memristor concept, their observed responses strongly resemble memristive inductance. However, they have not been interpreted within a memristive framework. The present work provides such an interpretation within a unified memristive framework. This points to a unifying principle: across diverse materials, slow internal dynamics coupled with hysteretic *I*-*V* behavior can manifest as colossal effective inductance.

Finally, realizing such enormous inductance values on-chip—without discrete coils—opens opportunities in low-frequency filtering, timing circuits, and neuromorphic computing. From a fundamental standpoint, it calls for theoretical exploration of the microscopic origin of memristive inductance, potentially linked to doublon–holon dynamics or nonequilibrium Mott transitions under bias^17^. Our findings thus redefine memristors not only as resistive-switching elements but also as intrinsic sources of emergent inductance—an overlooked property that significantly broadens the functional landscape of memristive devices.

## Conclusion

In summary, we have demonstrated that the q1D Mott insulator [Ni(chxn)$_2$Br]Br$_2$ functions as a memristor exhibiting a clear PHL under *ac* bias. This memristive behavior gives rise to a colossal emergent inductance of $10^4$–$10^5$ H, which appears only under finite bias and is independently confirmed by impedance spectroscopy and oscillation-frequency analysis.

We show that this inductive response is intrinsic to the memristive dynamics of the correlated molecular system and cannot be explained by conventional circuit elements or parasitic effects. The resulting interplay between emergent inductance and NDR enables self-sustained oscillations without any discrete inductor, providing a unified physical picture beyond conventional relaxation oscillator models.

These findings establish emergent inductance as a fundamental electrodynamic property of memristive systems and reveal a new functionality in correlated molecular materials. More broadly, they highlight how slow internal dynamics and hysteretic transport can generate unconventional circuit responses in solid-state systems, suggesting potential routes toward coil-free low-frequency functionalities.

## Methods

Single crystals of [Ni(chxn)$_2$Br]Br$_2$ were prepared following the reported procedure with slight modifications^18^. A solution containing 30 mM Ni(chxn)$_2$Br$_2$ and saturated tetramethylammonium bromide in anhydrous methanol was electrolyzed under a constant current of 20 $\mu$A using a Yazawa CS-12B constant-current source and 5-mm-diameter platinum wire electrodes. These electrodes were used only for crystal growth. After several days of electrolysis, black single crystals of [Ni(chxn)$_2$Br]Br$_2$ were obtained on the anode. The size of the single crystal used in this study was approximately 1.3 × 1.0 × 0.9 $\text {mm}^3$. The entire crystal contributes to the observed memristive transport behavior. The *ac* *I*-*V* characteristics (Lissajous curves) were obtained by applying a sinusoidal *ac* current from a Keithley 6221 source to the sample in series with a 10 k$\Omega$ load resistor. The *ac* current (*x*-axis of the Lissajous curve) was calculated from the voltage drop across the load resistor, while the *ac* voltage (*y*-axis) was measured across the sample. Impedance spectroscopy was performed using a frequency response analyzer (Solartron Analytical 1252 A) in combination with a dielectric interface (Solartron Analytical 1296). Cole–Cole plots were analyzed using the ZView software (Ametek Scientific Instruments). Memristive oscillation measurements were conducted by connecting external capacitors in parallel with the sample and applying a *dc* bias current $I_{bias}$ using the Keithley 6221. The oscillating voltage across the sample was monitored using an oscilloscope. All measurements were carried out using the same single-crystal sample with the conventional two-terminal configuration. The sample was mounted in a cryostat and cooled at a rate of 1 K/min.

## Supplementary Information

Supplementary Information.

## Data Availability

The data that support the findings of this study are available from the corresponding author upon reasonable request. Requests for materials should be addressed to Y.O. (yugo@riken.jp).
