# Supplementary material for: Colossal emergent inductance in a molecular memristor
Source: Sci Rep. 2026 May 8;16:13023. doi: 10.1038/s41598-026-48808-5 (PMC13156288; doi:10.1038/s41598-026-48808-5)
Supplement: Supplementary file 1 — Supplementary Information. [file 41598_2026_48808_MOESM1_ESM.pdf]

Supporting Information

Colossal emergent inductance in a Molecular Mott Memristor

Yugo Oshima[1], Rei Usami[2], Tetsuro Moriya[2], Taishi Takenobu[2] and Shinya Takaishi[3]*

The PDF file includes:

Supplementary Text
- Basic properties of $[Ni(chxn)_2Br]Br_2$
- Cole–Cole plots of R‖C, R‖L, R‖C‖L circuits, and memristor's equivalent circuits
- Additional impedance spectroscopy data
- Additional memristive oscillation data

Figure S1 to S5

Supplementary Text

Basic properties of $[Ni(chxn)_2Br]Br_2$

This study focuses on the quasi-one-dimensional (q1D) halogen-bridged mononuclear metal complex $[Ni(chxn)_2Br]Br_2$ (chxn = cyclohexanediamine).[1] Its crystal structure, shown in Fig. S1(a), features alternating linear chains of Br and Ni atoms. The Ni^{3+} ions adopt a $3d^7$ configuration with a half-filled $3d_{z^2}$ orbital that overlaps with the $4p_z$-orbitals of the bridging Br atoms. Strong on-site Coulomb repulsion splits the $3d_{z^2}$ band into lower and upper Hubbard bands, resulting in a Mott insulating ground state (Fig. S1(b)). As the $4p_z$-orbital lies within a Mott gap, the material is classified as a charge transfer-type Mott insulator. The energy gap between the valence and conduction bands is about 1.3 eV according to previous optical study.[2]

Figure S1(c) shows the temperature dependence of resistance along the chain axis. The resistance increases with decreasing temperature, confirming the insulating nature of the complex. The activation energy estimated from the Arrhenius plot is E_a=1287 K ~ 0.11 eV. The discrepancy from the optical gap (~1.3 eV) may arise from the in-gap states induced by impurities.[1a] The *I-V* characteristics (*dc*) becomes non-linear below 130 K, and the NDR feature starts to be observed below 105 K (Fig. S1(d)).

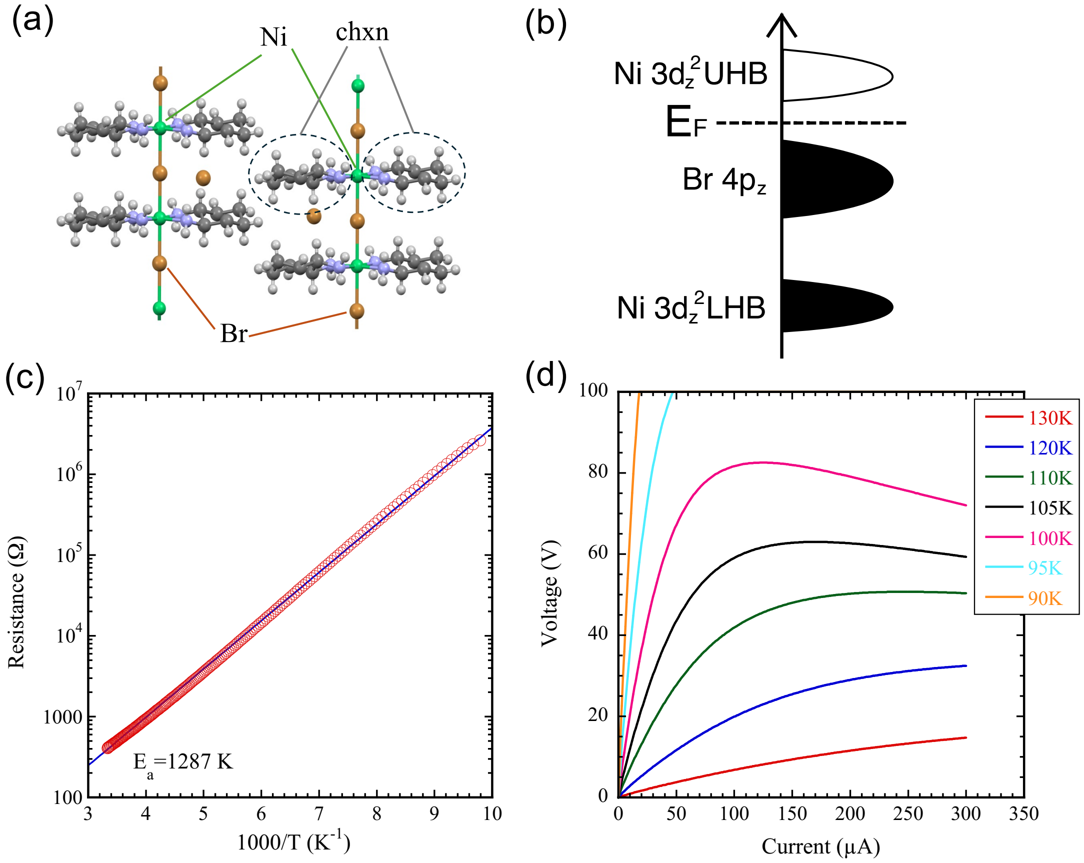

Figure S1.

(a) Crystal structure and (b) electronic band structure of $[Ni(chxn)_2Br]Br_2$. LHB and UHB stands for lower and upper Hubbard band, respectively. The temperature dependence of (c) resistance and (d) *I-V* characteristics along the chain-axis.

Cole–Cole plots of $R \| C$, $R \| L$, $R \| C \| L$, and memristor's equivalent circuits

The Cole–Cole plot, representing the imaginary versus real parts of complex impedance, serves as a fundamental tool for characterizing frequency-dependent electrical response in linear circuits.[3] In the case of simple parallel RC and RL circuits, the impedance response traces ideal semicircular arcs in the complex plane, reflecting their single relaxation dynamics. Below, we describe three characteristic shapes of Cole–Cole plots for parallel circuits, and the equivalent circuit for memristor.

(i) R and C in parallel ($R \| C$)
For the parallel RC circuit, the complex impedance is given by:

$$Z(\omega) = \frac{R}{1 + i\omega RC} = R\frac{1 - i\omega RC}{1 + (\omega RC)^2}.$$

Hence, the real and imaginary part of the impedance become

$$Re[Z] = \frac{R}{1 + (\omega RC)^2}, Im[Z] = -\frac{\omega R^2 C}{1 + (\omega RC)^2}.$$

By plotting Im[Z] versus Re[Z], one obtains a perfect semicircle located in the lower half of the complex plane. The center of the semicircle lies on the real axis at $R/2$, and its radius is also $R/2$. This shape arises because the impedance has a frequency-dependent imaginary part that is always negative (capacitive behavior), and it satisfies the equation of a circle:

$$\left(Re[Z] - \frac{R}{2}\right)^2 + (Im[Z])^2 = \left(\frac{R}{2}\right)^2.$$

(ii) R and L in parallel ($R \| L$)
For a resistor and inductor in parallel, the impedance is:

$$Z(\omega) = \frac{R}{1 - i\frac{R}{\omega L}} = R\frac{1 + i\frac{R}{\omega L}}{1 + \left(\frac{R}{\omega L}\right)^2}.$$

In this case, the Cole–Cole plot forms a semicircle in the upper half-plane, centered at $R/2$ with radius $R/2$, just like the previous RC case but reflected about the real axis. This is because the imaginary part of the impedance is always positive due to the inductive contribution. The same circular equation holds, but with $Im[Z] > 0$.

(iii) R, C, and L in parallel ($R \| C \| L$)
When a resistor, capacitor, and inductor are all connected in parallel, the total impedance becomes:

$$Z(\omega) = \frac{1}{R} + i\omega C + \frac{1}{i\omega L}.$$

This circuit exhibits both capacitive and inductive behavior depending on the frequency. At low frequencies, the inductive branch dominates, and the Cole–Cole plot lies in the upper half-plane (inductive). At high frequencies, the capacitive branch dominates, and the plot shifts to the lower half-plane (capacitive). Near the resonant frequency $\omega_0 = 1/\sqrt{LC}$, the imaginary part of the admittance vanishes, and the plot crosses the real axis. As a result, the Cole–Cole plot for the $R \| C \| L$ circuit exhibits a continuous curve that crosses the real axis and smoothly transitions from an inductive arc in the upper half-plane to a capacitive arc in the lower half-plane. While the shape may resemble two connected semicircles, the overall response deviates from ideal Debye-type relaxation and instead reflects the coexistence and competition of multiple reactive pathways within the circuit.

(iv) Memristor's equivalent circuit

The equivalent circuit of memristor can be found in Fig. S2(a). It consists of two resistors (R_{S1} and R_{S2}), and parallel L and C elements. Similar to the simple $R\|C\|L$ circuit, the Cole-Cole plot in the low-frequency range is dominated by the inductive semicircle, and the high-frequency range is dominated by the capacitive semicircle as shown in Fig. S2(b). The left intercept of the inductive semicircle corresponds to the low-frequency limit of the real impedance, representing the slope resistance R_{S1}. The right intercept indicates the high-frequency limit, corresponding to the static resistance (R_{S1}+R_{S2}).

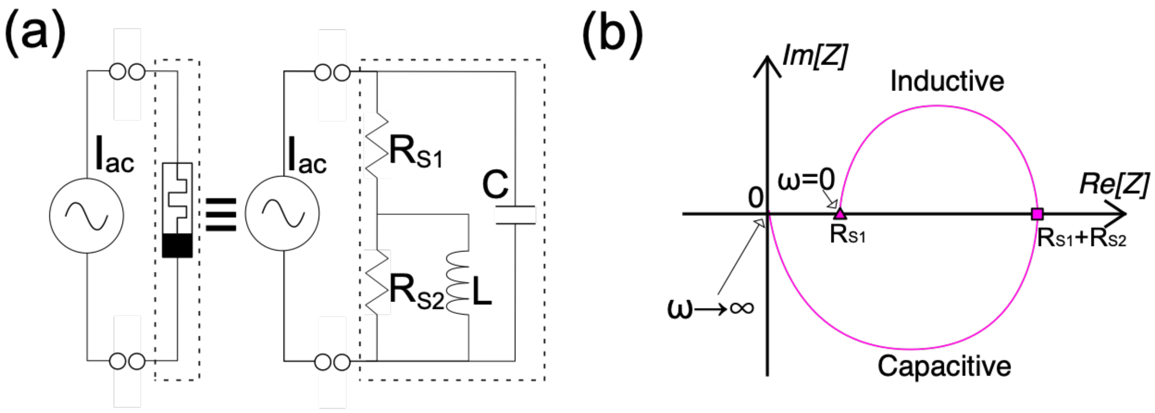

Figure S2.
(a) Equivalent circuit of the Mott memristor, and (b) its expected Cole-Cole plot.

Additional impedance spectroscopy data

Additional temperature-dependent impedance spectroscopy results are shown in Fig. S3. At V_{bias} = 0 V, the Cole–Cole plots exhibit purely capacitive behavior, which can be well reproduced by the equivalent circuit shown in Fig. 3(b) with R_{S2}=0 and L=0. The extracted parameters R_{S1} and C are plotted in Fig. S3(b). R_{S1} increases markedly as temperature decreases, while C remains nearly constant at ~0.1 nF. These results are consistent with the transport measurements (Fig. S1(c)) and support the robustness of the equivalent-circuit analysis

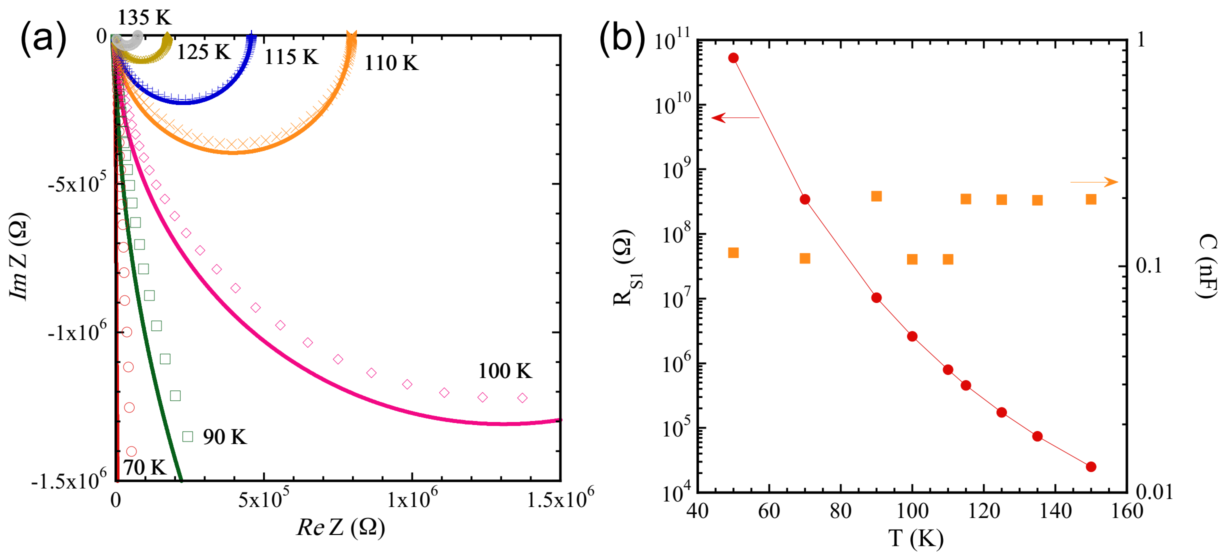

Figure S3.
(a) Temperature dependence of the Cole–Cole plots at V_{bias} = 0 V, with corresponding fitted curves using the same equivalent circuit as in Fig. 3(b). The applied *ac* voltage was 3 V. For the 70–100 K data, the high-impedance region is outside the plotted range for clarity. (b) R_{S1} and C as functions of temperature, extracted from the fits in (a).

Additional memristive oscillation data

Figure S4(a) presents the bias-current dependence of memristive oscillations at 99 K with an external capacitor C = 4.9 μF. In Fig. S4(a), when the applied bias current (I_{bias}) exceeds the threshold of ~160 μA, self-sustained oscillations emerge. The oscillation amplitude increases and the oscillation frequency shifts slightly upward with I_{bias}. The corresponding FFT spectra in Fig. S4(b) confirm the increase of fundamental frequency with I_{bias}.

Figure S5(a) shows the capacitance dependence of memristive oscillations at 103.5 K. The oscillation frequency systematically increases as the external capacitance is reduced (C = 10, 4.9, and 1 μF), in accordance with the LC resonance condition. However, when the oscillation frequency exceeds ~0.3 Hz, the oscillations become damped and eventually disappear (Fig. S5(b)). This behavior is consistent with the frequency–temperature dependence of the PHL and NDR features (see Fig. 2 of the main text), confirming that NDR is essential to sustain oscillation.

These additional data further reinforce that the observed oscillations arise from the interplay of emergent inductance and NDR in $[Ni(chxn)_2Br]Br_2$, rather than from parasitic circuit elements.

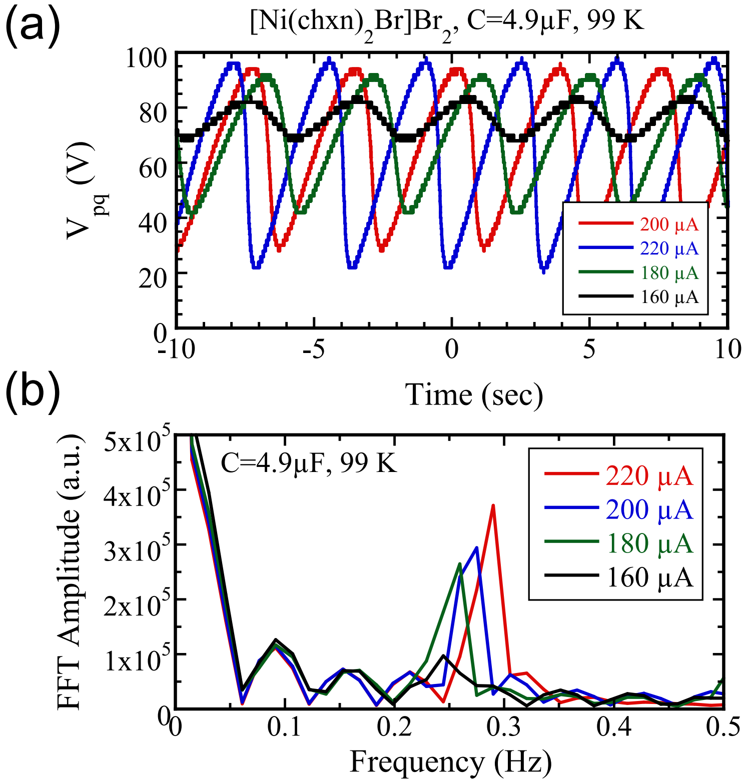

Figure S4.
Typical memristive oscillation at 99 K with different sets of bias current I_{bias}=160, 180, 220 and 220 μA. The oscillation circuit diagram is shown in Fig. 5(e), and external capacitor C=4.9 μF is used. (b) FFT spectra of the memristive oscillation observed in (a).

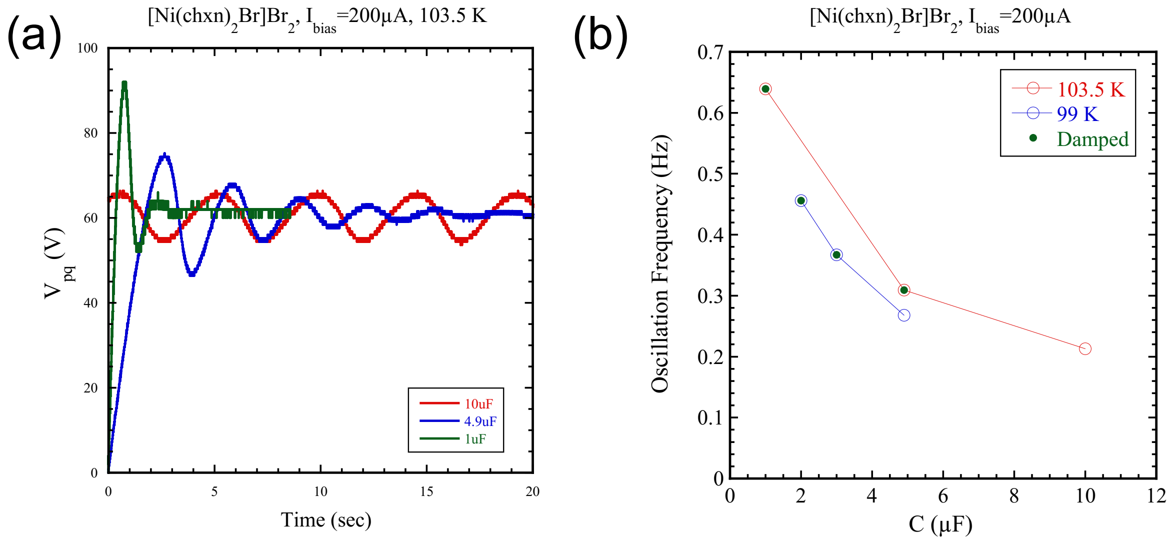

Figure S5.

(a) Memristive oscillation using different sets of external C=1.0, 4.9, and 10 μF measured at 103.5 K. I_{bias}=200 μA is applied to the memristive oscillation's circuit. The oscillation circuit diagram is shown in Fig. 5(e). (b) Capacitance dependence of the oscillation frequency. Although the operating frequency increases with decreasing C, the oscillation damps when the oscillation frequency exceeds ~0.3 Hz.

References

[1] a)K. Toriumi, Y. Wada, T. Mitani, S. Bandow, M. Yamashita, Y. Fujii, *J. Am. Chem. Soc.* **1989**, 111, 2341; b)H. Okamoto, K. Toriumi, T. Mitani, M. Yamashita, *Phys. Rev. B* **1990**, 42, 10381; c)H. Kishida, H. Matsuzaki, H. Okamoto, T. Manabe, M. Yamashita, Y. Taguchi, Y. Tokura, *Nature* **2000**, 405, 929; d)S. Iwai, M. Ono, A. Maeda, H. Matsuzaki, H. Kishida, H. Okamoto, Y. Tokura, *Phys. Rev. Lett.* **2003**, 91.

[2] H. Okamoto, Y. Shimada, Y. Oka, A. Chainani, T. Takahashi, H. Kitagawa, T. Mitani, K. Toriumi, K. Inoue, T. Manabe, M. Yamashita, *Phys. Rev. B* **1996**, 54, 8438.

[3] J. R. Macdonald, *Impedance Spectroscopy - Emphasizing Solid Materials and Systems*, John Wiley & Sons, Inc., **1987**.
